# Pneumorrhachis Following Neck Penetrating Injury: A Case Report

**DOI:** 10.7759/cureus.31925

**Published:** 2022-11-26

**Authors:** Lamiz Tannouri, Omar Q Muhammed Noori, Yousif Habib Hussain Nasir Alabboudi, Hayder S Abdulhadi, Maher Younes Alobeid

**Affiliations:** 1 Emergency Medicine, Dubai Health Authority (DHA), Dubai, ARE; 2 Emergency Medicine, Rashid Hospital Trauma Center, Dubai Health Authority (DHA), Dubai, ARE; 3 General Surgery, Rashid Hospital Trauma Center, Dubai Health Authority (DHA), Dubai, ARE; 4 Orthopedics, Rashid Hospital, Dubai, ARE

**Keywords:** head and neck trauma, neck, free air on spinal canal, pneumocele, penetrating neck injury, pneumorrhachis

## Abstract

The development of air in the spinal canal is an uncommon and usually asymptomatic event. Also known as pneumorrhachis (PNR), the main information about this phenomenon is based on a few case reports published previously. It is highly difficult to identify this entity clinically, and in most publications, PNR was incidentally identified during image procedures, mainly computed tomography (CT) scans. With the advancement of technology and the development of guidelines for the treatment of penetrating and neck injuries, the number of PNR diagnosis has increased. It is also a common agreement among the articles reviewed that the least common cause of PNR is traumatic events. This report presents a rare case of pneumorrhachis as a consequence of a penetrating neck injury. The studied patient was a 27-year-old female with multiple stab wounds on the left posterior side of the neck and left shoulder, thereby developing left-side body weakness as a consequence of the wound. The patient was immediately evaluated and managed by the emergency team, and as the patient was vitally stable, she was shifted to an urgent CT scan. CT scan showed subarachnoid air focus, multiple extradural air foci, and spinal cord injury on the cervical spine. This patient was treated conservatively, but her neurological symptoms persisted until discharge.

## Introduction

Pneumorrhachis (PNR), also known as a spinal canal filled with air, is a rare radiological finding. This is usually asymptomatic and underdiagnosed in patients who are exposed to physical trauma. It has been increasingly identified in recent past years through better trauma guidelines and improved imaging modalities, especially computed tomography (CT) [1,2].

We present a case of a female patient who was stabbed in the neck and shoulder and developed neurological weakness on the left side of her body. CT scan and magnetic resonance imaging (MRI) images showed the presence of subdural and epidural PNR and evidence of spinal cord injury. Furthermore, we will also review the history and findings of the disease with the most common causes, symptoms, and complications as well as the best ways to approach it according to the most updated literature.

## Case presentation

A 27-year-old female presented to the emergency department (ED) with a history of multiple stab wounds on the left posterior side of the neck and left shoulder. The patient was stabbed around 30 minutes before arrival by a kitchen knife of unknown size. Upon arrival, she was conscious and oriented, complaining of left-side body weakness on both upper and lower limbs. The patient was then immediately shifted to the resuscitation room (RR) for primary assessment. As per the assessment, it was found that the airway was patent, and breathing was equal and clear to both lungs, with SpO_2_ of 98% on room air. Further, pulses were normal and equal in all limbs, blood pressure (BP) was 90/60 mmHg, and heart rate (HR) was 98 beats per minute (bpm). The neurological assessment showed a Glasgow Coma Scale of 15/15, and both pupils were equally reactive. Left upper and lower limb power was 3/5 and 0/5, respectively. The sensation was intact in all limbs.

Upon examination, no obvious deformities and no signs of blunt trauma were found. A deep lacerated wound of approximately 5-6 cm on the posterior aspect of the left side of the neck on zone 2 was present, with a clear transection of the platysma muscle. In addition, there were three lacerated wounds on the left upper back measuring around 3, 4, and 7 cm, respectively. No active bleeding was visualized. On log roll, no deformity and no midline tenderness were observed. Moreover, extended focus assessment with sonography for trauma (E-fast) showed neither signs of hemothorax or pneumothorax nor pericardial effusion or free intraperitoneal fluid.

The patient was immediately managed by the emergency team and kept on a cardiac monitor and close observation. Further, analgesics, intravenous fluids, and tetanus toxoid booster were also administered.

**Figure 1:**
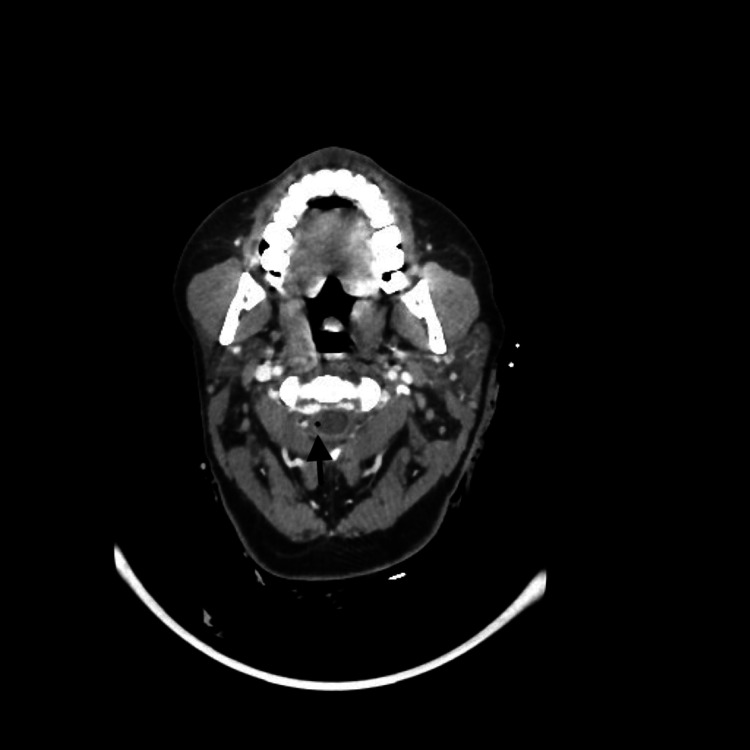
CT image showing intrathecal air focus at the C2-C3 level on the right side

**Figure 2:**
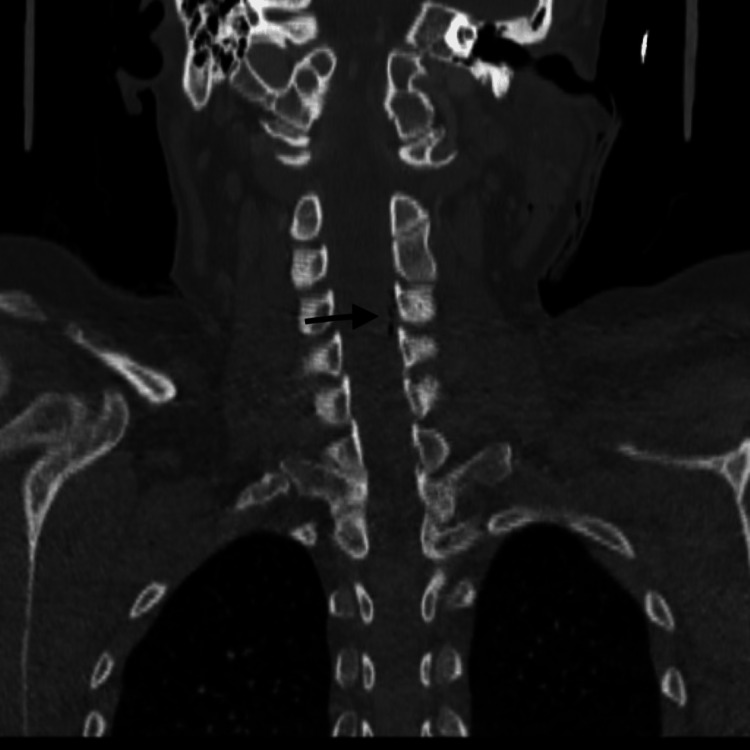
CT image showing multiple extradural air foci on the left cervical spinal from C4 to C6 level

An urgent contrast CT of the patient’s neck was done as she had stable vital signs. CT results showed intrathecal air focus at C2-C3 level on the right side, multiple extradural air foci on the left cervical spinal from C4 to C6 (Figures 1, 2), and nondisplaced fracture of the left lamina of C3. Hemorrhagic foci in the left trapezius muscle with intermuscular fluid and surgical emphysema in the left posterior paraspinal space were also visualized on the CT, without evidence of main vessel transection on the images.

**Figure 3:**
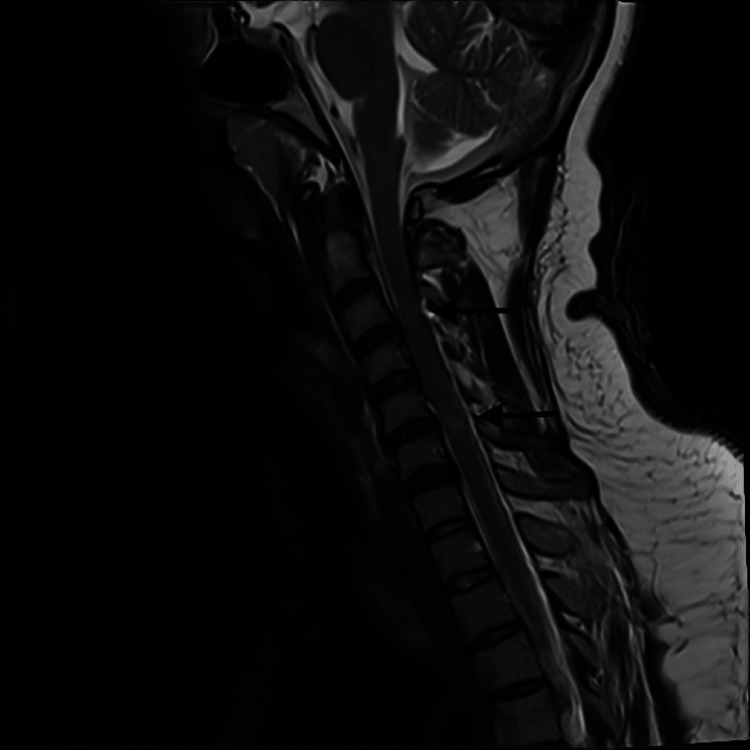
MRI image showing spinal cord injury at C3-C4 and C5-C6 levels

The patient’s general condition and laboratory parameters remained stable during ED evaluation. Due to the deep wound (penetrating platysma muscle) in the neck, the general surgery team explored and repaired the wound in the operating room, and the patient was put on prophylactic antibiotics. The team concluded that no main vessels were damaged by the injuries, and bleeding was mainly from minor branch vessels. No cerebrospinal (CSF) leak was identified from her neck wound. General surgery and neurosurgery teams handed over the case to the trauma/orthopedic team as no further acute intervention is needed. And the case was admitted by the trauma/orthopedic team for further workup and management. During admission, the patient underwent an MRI of the spine, which showed spinal cord injury at C3-C4 and C5-C6 levels (Figure 3) and defects of ligamenta flava and posterior dura at C3-C4 levels. No sizable intraspinal hematoma was reported.

The patient's neurological symptoms persisted with no improvement. On admission, she was put on a Philadelphia c-collar and then shifted to a soft neck collar before discharge. As the patient's fracture and clinical status remained stable despite the persistence of the neurological deficits, she was discharged with clinic follow-up. However, the patient did not attend the clinic appointments.

## Discussion

The term "pneumorrhachis" refers to the presence of free air in the spinal canal and represents an uncommon phenomenon that is usually asymptomatic and originally underdiagnosed. As radiology techniques advanced through the years, this pathology has been more frequently identified [1,2]. It was first described by Gordon and his coworkers in 1977 by the entity of pneumomyelogram. In 1987, Newbold and his team were the first to define air in the vertebral column of a trauma patient as PNR [3,4]. Throughout the medical literature, many other terms have been used to describe this same condition, such as intraspinal pneumocele or pneumocoele, spinal epidural and subarachnoid pneumatosis, spinal and epidural emphysema, and many others [2,4].

PNR knowledge is based mainly on case reports and short case series published. Oertel et al. conducted a comprehensive literature review of the U.S. National Library of Medicine’s Medline bibliographic database till December 2005, which revealed 71 articles that reported on 86 cases only, involving traumatic and nontraumatic causes [2].

It is highly difficult to identify this problem clinically. In most publications, PNR was incidentally identified during imaging procedures by plain radiography, CT, or MRI [4]. CT scan is the preferred imaging technique for diagnosis, with 100% sensitivity for diagnosing PNR [1,5]. Similarly, in the presented case, PNR was first visualized on the CT scan and further analyzed with the MRI.

The occurrence of PNR has been correlated with a variety of causes that could be classified as iatrogenic, traumatic, and nontraumatic [4,5]. However, the most common causes are iatrogenic, with epidural emphysema being a well-known complication of epidural analgesia. The presence of PNR caused by traumatic events is extremely rare. A literature review performed by Goh et al. till April 2005 revealed only 31 reported cases of traumatic PNR [4].

It is also important to highlight that the most common causes of traumatic PNR include injury from road traffic accidents and heightened falls [3,4]. A systematic review by Osunronbi et al. analyzed 82 case reports of traumatic PNR published up to June 2019, with 96 individual cases. They report that 80% of patients had blunt trauma, while 17% had penetrating injuries [1]. One of the main reasons behind the increase in the diagnosis of PNR is the development and implementation of guidelines in the workup of penetrating and blunt neck injuries such as the Biffl criteria in the workup for blunt cerebrovascular injuries [6].

When investigating traumatic PNR, distinguishing between air in the epidural and subarachnoid space is extremely important [4,5,7]. As each presentation might be associated with different causes, pathophysiologic mechanisms, and clinical features, and because it is well established that while epidural air is usually benign, air in the subarachnoid space is a marker of major trauma and severity, often associated with tension pneumocephalus or meningitis [4,5]. Goh et al. also reported that from the 31 cases, 18 were reported with air in the subarachnoid space. Of those, 12 cases had associated skull fractures, and five cases had thoracic spine fractures. It was also observed that 77.8% of those 18 cases had associated pneumocephalus [4].

As PNR is a rare condition with different pathogenesis and etiologies, there are no empiric guidelines or standards of care for it [2,5,7]. Generally, PNR is asymptomatic and spontaneously resorbs after several days. In some circumstances, this condition might be symptomatic, causing pain or neurologic deficits. Some cases might even require surgical decompression or repair, mostly related to entrapped intraspinal air under pressure entering the craniospinal compartment in combination with a one-way air valve mechanism, causing tension PNR and pneumocephalus with nervous tissue compression [2,5].

A case report published by Kara et al. in the *American Journal of Emergency Medicine* showed two patients who presented with penetrating injuries on the posterior aspect of the neck and muscle weakness restricted to one side of the body. PNR was diagnosed incidentally on CT scans, and both cases demonstrated pneumocephalus on the CT brain. One of the patients was later diagnosed with persistent cerebral fluid drainage and was treated with surgical repair of the dural laceration. Both patients were discharged with no major neurologic sequelae [5].

In our case, a young female presented with a 5-6-cm stab wound on the left posterior aspect of the neck, specifically on zone 2. She was conscious and oriented, complaining of left-side body weakness, with power 3/5 in the left upper limb and 0/5 in the left lower limb. The CT scan showed subdural air focus at the C2-C3 level on the right side and multiple extradural air foci on the left cervical spinal from C4 to C6. MRI was also performed and showed spinal cord injury at C3-C4 and C5-C6 levels as well as defects of ligamenta flava and posterior dura at C3-C4 levels. The patient underwent wound exploration, which concluded that no major vessels were damaged, and there were no signs of CSF leakage.

In comparison to the previous case reports that stated most patients have an improvement in neurological function post PNR, our patient did not demonstrate any improvement in neurologic function until the moment of discharge. However, according to our spine orthopedic trauma specialist, this patient clearly developed Brown-Séquard syndrome because of the penetrating injury to the spinal cord and not due to the PNR. The PNR is a rare entity, but most case reports clearly state that it is usually asymptomatic and incidentally diagnosed. The conservative management is adequate as the air will be reabsorbed and disappear, without any other complications.

## Conclusions

PNR is an uncommon and frequently undiagnosed presentation. The literature is mostly based on case reports. The clinical diagnosis of this pathology is highly difficult as the majority of patients are asymptomatic, with most PNR cases being incidentally identified on image studies, with CT scans being the preferred technique. The literature review also highlights that penetrating injuries are a rare cause of PNR. Although most cases are treated conservatively with the improvement of neurological function, it is important to diagnose PNR and differentiate between the subdural and extradural, due to the risks of complications, such as pneumocephalus.

This article described a rare case of penetrating neck injury who presented as vitally stable with left-side body weakness. CT scan of the cervical spine showed the presence of subdural and extradural PNR. This patient was treated conservatively, but her neurological symptoms persisted most likely due to the spinal cord injury.
